# Identification and Characterization of the MAPK Genes in Sorghum and Their Expression in Response to Aphid Infestation

**DOI:** 10.3390/plants15213342

**Published:** 2026-10-28

**Authors:** Saima Sadia, Kumar Shrestha, Andrew N. Doust, Yinghua Huang

**Affiliations:** 1 Department of Biology, Oklahoma State University, Stillwater, OK 74078, USA; 2 Plant Science Research Laboratory, United States Department of Agriculture-Agricultural Research Service (USDA-ARS), Stillwater, OK 74075, USA

**Keywords:** aphid, biotic stress, insect resistance, mitogen-activated protein kinase, signaling molecule, *Sorghum bicolor*

## Abstract

Mitogen-activated protein kinase (MAPK) cascades are key signaling components that regulate plant growth, development and responses to stresses. In this study, 19 sorghum MAPK genes (*SbMAPKs*) were identified through comprehensive bioinformatic analyses, including sequence alignment, gene structure characterization, conserved motif analysis and phylogenetic analysis. Phylogenetic analysis classified the *SbMAPK* family into five subfamilies (A–E). Evolutionary analyses suggested that tandem duplication, along with strong purifying selection, contributed to the expansion and conservation of the *SbMAPK* gene family in sorghum. Promoter analysis revealed that most *SbMAPKs* contain predicted cis-regulatory elements associated with phytohormone signaling and biotic and abiotic stress responses. Furthermore, quantitative real-time PCR (qRT-PCR) analysis identified nine *SbMAPK* genes that were differentially expressed following sorghum aphid infestation. Among these, *SbMAPK4* consistently exhibited higher expression in the resistant genotype, suggesting a potential role in aphid-induced defense responses. These findings identify candidate *SbMAPKs* for functional characterization and provide new insights into the evolution of the MAPK gene family, laying the groundwork for elucidating the molecular mechanisms underlying aphid resistance and supporting the genetic improvement of sorghum.

## 1. Introduction

Sorghum (*Sorghum bicolor* (L.) Moench), a C4 grass known for its excellent drought tolerance, serves various purposes including the following: it is a staple food, it can be foraged and it is a renewable source of biofuel [1]. It is the fifth-most significant cereal crop in the world after maize, rice, wheat and barley [2]. The sorghum aphid, previously referred to as the sugarcane aphid, *Melanaphis sacchari* (Zehntner, 1897), is now an important pest in sorghum cultivation areas [3]. It reduces grain quality [4] and can cause a significant yield loss of 30% to 100% [3]. Several varieties are known to be resistant to aphids, but the molecular mechanism underlying their resistance remains unclear.

Plant defense against insects largely depends on perceiving the invading insects, followed by a reaction to them and finally a transduction of that signal into defense responses. Its responses to biotic and abiotic stresses are initiated by protein kinases, which are crucial in signal transduction pathways [5]. The signaling molecules, such as MAPKs, are among the first components in the interaction between host plants and attacking insects [6]. When herbivores attack the plant, the MAPKs send out a signal, and subsequently phytohormone biosynthesis is activated, which transforms the downstream gene expression, i.e., transcriptome and proteome, for self-protection [7]. The common MAPK cascade consists of three classes of interconnected protein kinases: MAPK kinase kinase (MAPKKKs/MEKKs), MAPK kinase (MAPKKs/MKKs) and MAPK [8]. These three types of protein kinases function as sequential signal transducers and deliver information by means of phosphorylation. MAPKKKs activate MAPKs by phosphorylating the threonine and tyrosine residues of a TXY motif in the activation loop [9]. The activated MAPKs then regulate the genetic program of the downstream genes that allow plants to react to biotic and abiotic stresses by interacting with various effector proteins in the cytoplasm or nucleus [10].

The MAPKs harbor 11 conserved protein kinases, with TXY phosphorylation motifs in the activation loops between kinase subdomains VII and VIII, which provide the protein-binding domains necessary for MAPK activation. Based on TXY motifs, the MAPKs can be divided into four subfamilies, A, B, C and D. All four subfamilies have been reported to have a defense function [9]. With the completion of numerous plant genome sequencing projects, many members of the MAPK cascades have been identified and characterized in various plant species. Plant genome sequencing has identified 20 MAPKs in Arabidopsis [9,10], 19 in maize [6], 17 in rice [11], 20 in wheat [12], 20 in barley [13], 16 in *Brachypodium* [14], 16 in tea [15], 25 in poplar [16] and 16 in foxtail millet [17]. Across species, members of the same subfamily share conserved motifs and exon–intron structures, suggesting conserved biological functions [15].

Although there are a few reports about MAPKKs and MAPKKKs in sorghum, no sorghum MAPKs have been studied, particularly their responses to aphid infestation. It is not known which *SbMAPKs* respond to aphid feeding, or whether their responses differ between resistant and susceptible sorghum genotypes. In this study, we re-examined the MAPK gene family in the current sorghum genome and characterized its members in terms of phylogeny, gene structure, conserved motifs, gene duplication, and synteny with rice and *B. distachyon*. We predicted cis-regulatory elements and candidate transcriptional regulators in *SbMAPK* promoters and tested the predicted regulators for co-expression with *SbMAPKs* in our aphid-infestation transcriptome. Finally, we measured *SbMAPK* expression by qRT-PCR in the resistant genotype Tx2783 and the susceptible genotype BTx623 during sorghum aphid infestation. The results revealed some *SbMAPKs* with genotype- and time-dependent responses to aphid feeding as candidates for functional studies of aphid resistance.

## 2. Results

### 2.1. Identification of MAPK Genes in Sorghum

Protein sequences of *Brachypodium distachyon* were used as queries for BLAST analsis in the Phytozome database (https://phytozome-next.jgi.doe.gov/ 10 January 2023) against the *Sorghum bicolor* genome (version v3.1.1), resulting in the initial identification of 325 putative MAPK sequences. Candidate sorghum MAPK sequences were aligned with *B. distachyon* MAPK proteins, and the conserved TXYxxTRWYRAPE motif was manually examined. Candidate sequences were subsequently subjected to InterPro domain analysis, which confirmed 19 MAPK genes in the sorghum genome based on the presence of the conserved MAPK domain (PF00069) (Table 1).

The encoded *SbMAPK* proteins ranged from 275 amino acids (*SbMAPK3*) to 614 amino acids (*SbMAPK4*), with molecular weights of 42.4 to 70.0 kDa and isoelectric points of 5.31 to 9.66 (Table 1). Most *SbMAPK* proteins were predicted to localize to the cytoplasm (12 of 19), with the remainder predicted in the nucleus (4) or chloroplast (3) (Table S1).

### 2.2. Phylogenetic Relationship and Motif Analysis

**Figure 1:**
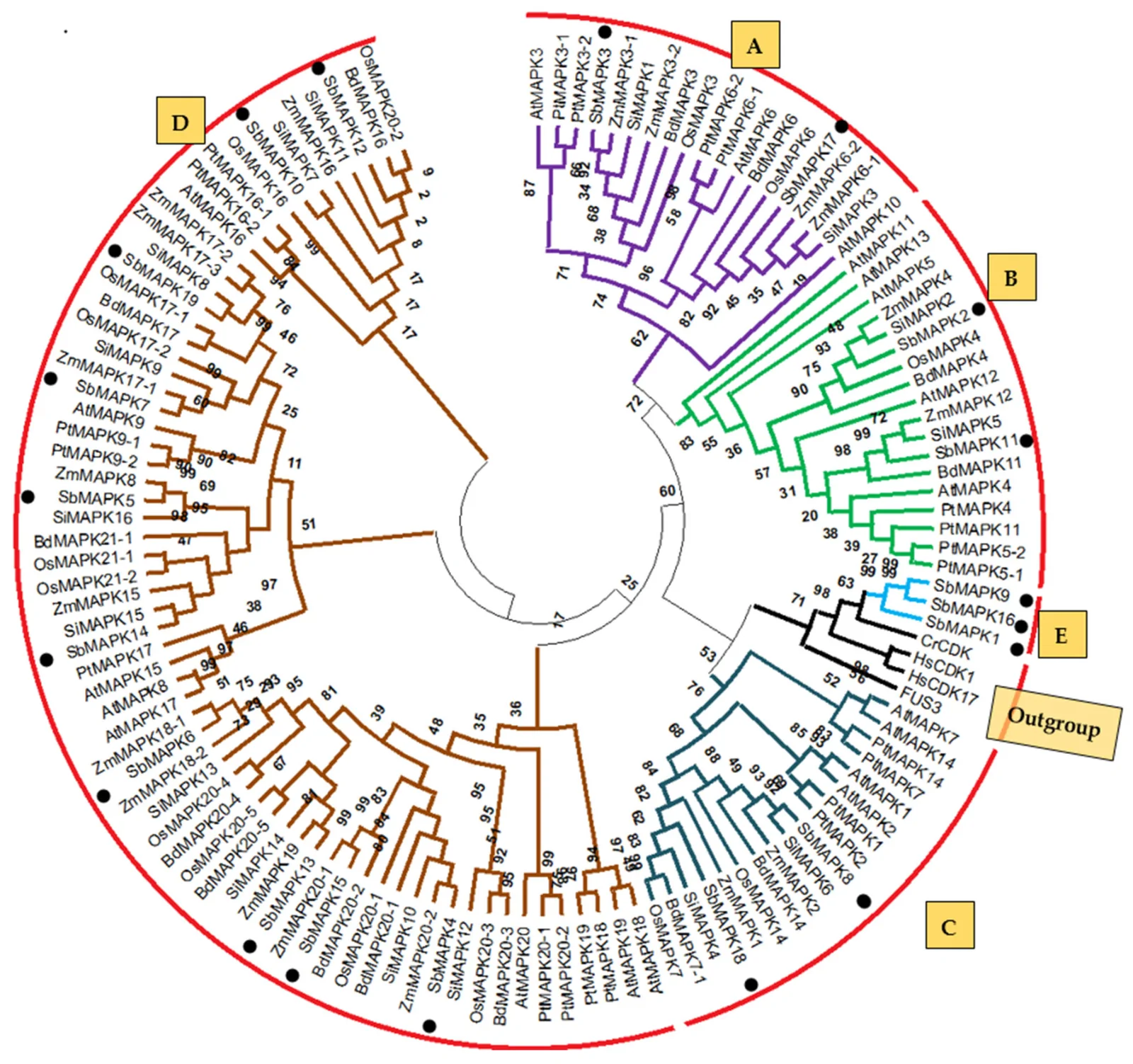
Phylogenetic relationships of the MAPK gene family. The maximum likelihood tree was constructed with MEGA 11 using the Jones–Taylor–Thornton (JTT) substitution model and 1000 bootstrap replicates, based on MAPK protein sequences from *Sorghum bicolor* (*Sb*), *Arabidopsis thaliana* (*At*), *Oryza sativa* (*Os*), *Zea mays* (*Zm*), *Setaria italica* (Si), *Brachypodium distachyon* (*Bd*), *Populus trichocarpa* (*Pt*), *Chlamydomonas reinhardtii cyclin-dependent kinases* (*CrCDK*), *Saccharomyces cerevisiae Fus3 protein* (*Fus3*) and *Homo sapiens cyclin-dependent kinases 1* and *17* (*HsCDK1* and *HsCDK17*). Subfamilies A–E are indicated by colored branches; black dots mark the *SbMAPK* proteins. Also, outgroups are shown in black colored branches. Subfamilies are shown by branch color: A, purple; B, green; C, dark teal; D, brown; E (MAPK-like proteins), light blue; outgroup, black. Red arcs around the tree mark the subfamily boundaries, numbers on the branches are bootstrap values (%), and black dots mark the *SbMAPK* proteins.

**Figure 2:**
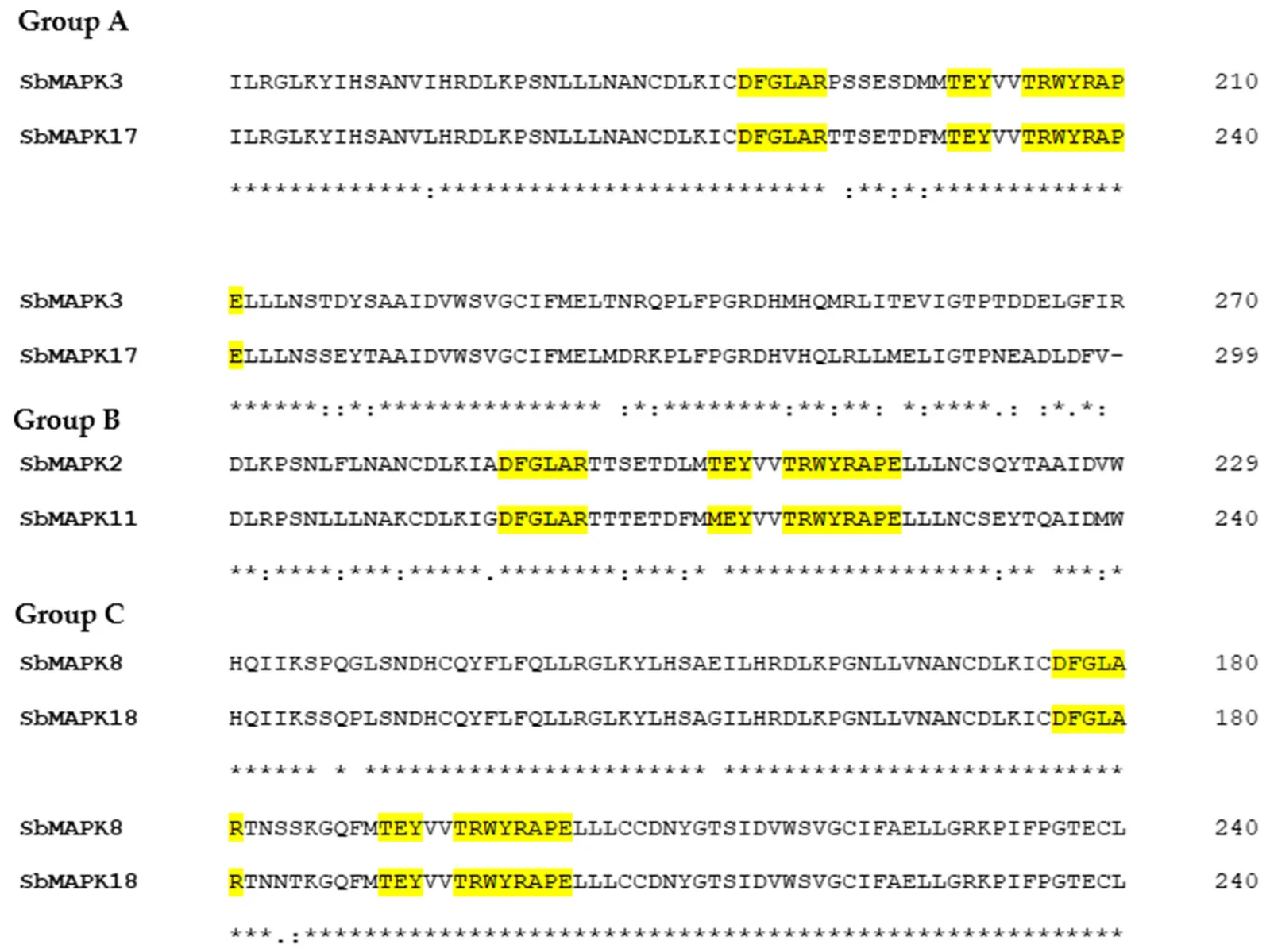

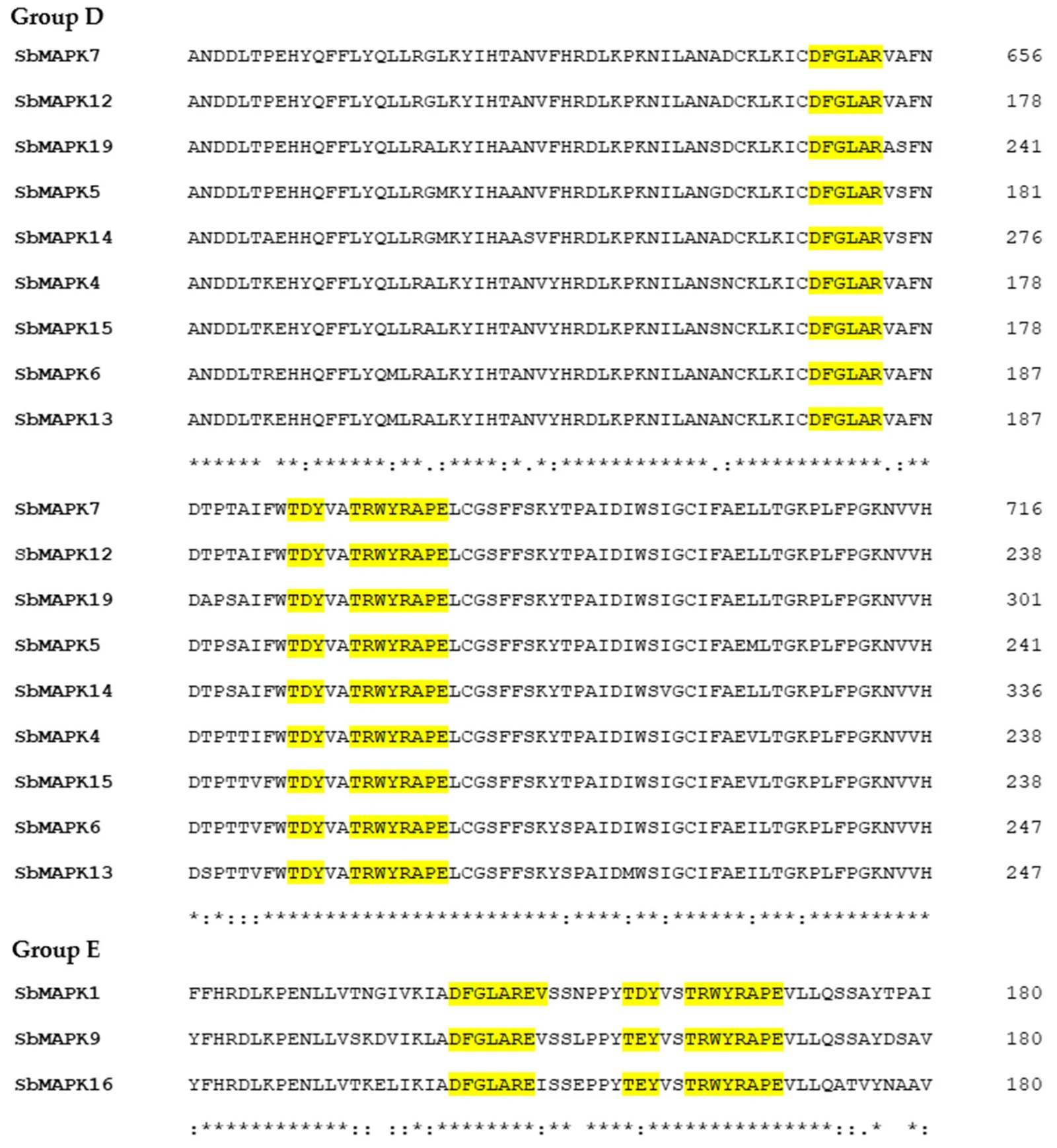
Multiple sequence alignment of the activation-loop region of sorghum MAPK proteins. (**A**) Subfamily A; (**B**) subfamily B; (**C**) subfamily C; (**D**) subfamily D; (**E**) subfamily E (MAPK-like proteins). The conserved DFGLAR motif and the TXYxxTRWYRAPE signature, which contains the TXY phosphorylation motif, are highlighted in yellow. Numbers on the right indicate amino acid positions. Asterisks, colons and periods below the alignment indicate fully conserved, strongly similar and weakly similar residues, respectively.

To classify the *SbMAPKs*, a maximum likelihood phylogenetic tree with MAPK proteins from *Arabidopsis thaliana*, *Oryza sativa*, *Zea mays*, *Setaria italica*, *Brachypodium distachyon* and *Populus trichocarpa*, using human *cyclin-dependent kinases* (*HsCDK1* and *HsCDK17*), Chlamydomonas reinhardtii cyclin-dependent kinases (*CrCDK*), Chlamydomonas reinhardtii cyclin-dependent kinases (*CrCDK*) and the yeast MAPK *FUS3* as an outgroup (Figure 1). Sixteen *SbMAPKs* clustered into four canonical plant MAPK subfamilies: subfamily A (*SbMAPK3* and *SbMAPK17*), subfamily B (*SbMAPK2* and *SbMAPK11*), subfamily C (*SbMAPK8* and *SbMAPK18*) and subfamily D (*SbMAPK4*, *SbMAPK5*, *SbMAPK6*, *SbMAPK7*, *SbMAPK10*, *SbMAPK12*, *SbMAPK13*, *SbMAPK14*, *SbMAPK15* and *SbMAPK19*). Each subfamily contained Arabidopsis, poplar and grass MAPKs, and *SbMAPK3* and *SbMAPK17* grouped with the *MAPK3* and *MAPK6* orthologs of the other species, respectively. The conserved TRWYRAPE and DFGLAR motifs were present in all 19 proteins (Figure 2).

Subfamily A and C members contained the TEY phosphorylation motif, and all subfamily D members contained the TDY motif. In subfamily B, *SbMAPK2* contained TEY, whereas *SbMAPK11* contained MEY. *SbMAPK11* grouped with *BdMAPK11*, an *MPK11* ortholog that also carries the MEY motif, and MEY is likewise present in the MPK11 orthologs of Triticeae species [18]. In contrast, group E, *SbMAPK1* (TDY), *SbMAPK9* (TEY) and *SbMAPK16* (TEY), did not group with any plant MAPK subfamily but formed a separate cluster next to the outgroup kinases (Figure 1). These three proteins also differed from the canonical *SbMAPKs* in the activation-loop region (Figure 2), and we therefore classified them as MAPK-like proteins.

The conserved ‘TRWYRAPE’ and ‘DFGLAR’ motifs were identified in 19 *MAPKs* (Figure 2). Subfamily A contains phosphorylation motif TEY; subfamily B contains both TEY and MEY; C members possess TEY and TDY; and subfamily D members feature the TDY motif at the activation site.

### 2.3. Conserved Motifs, Chromosomal Distribution and Gene Structure

**Figure 3:**
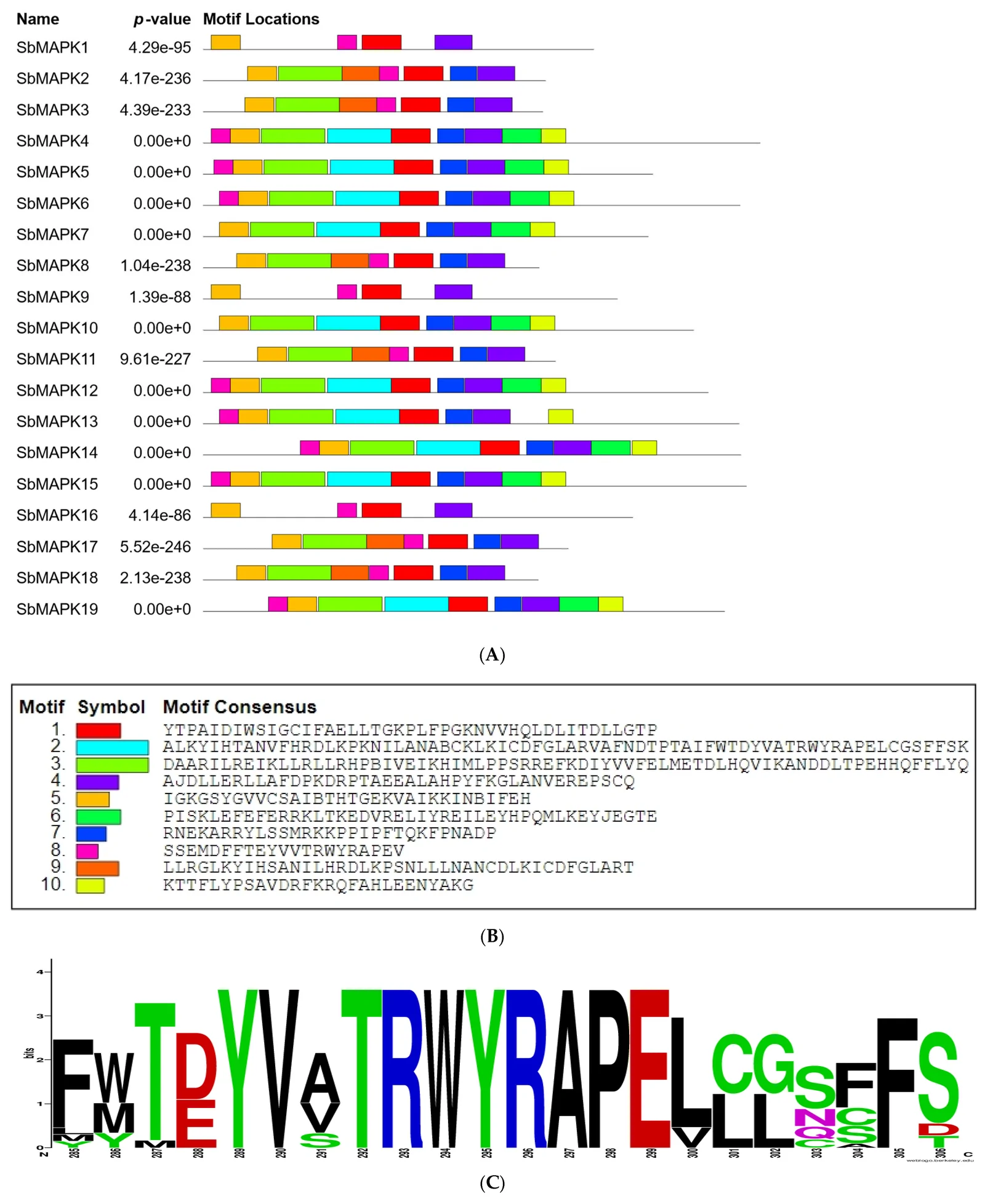
Conserved motifs of the *SbMAPK* proteins. (**A**) Distribution of the ten conserved motifs identified with MEME in each *SbMAPK* protein. Colored boxes represent individual motifs, and the combined *p*-value for each protein is shown next to its name. (**B**) Consensus sequences of the ten motifs. (**C**) Sequence logo of the activation-loop region containing the TXY phosphorylation motif; the height of each letter indicates the conservation of that amino acid at each position.

Ten distinct conserved motifs were identified within the *SbMAPK* protein family using the MEME Suite (http://meme-suite.org/tools/meme, 4 October 2026) (Figure 3A,B). Of these, motif 8 (TXYxxTRWYRAPE; pink box) was consistently detected in all 19 *SbMAPK* proteins, although its position varied among proteins. Sequence logos generated using the WebLogo online tool further confirmed the conservation of key residues within the *SbMAPK* proteins, with the TXY motif consistently present in all nineteen members (Figure 3C).

**Figure 4:**
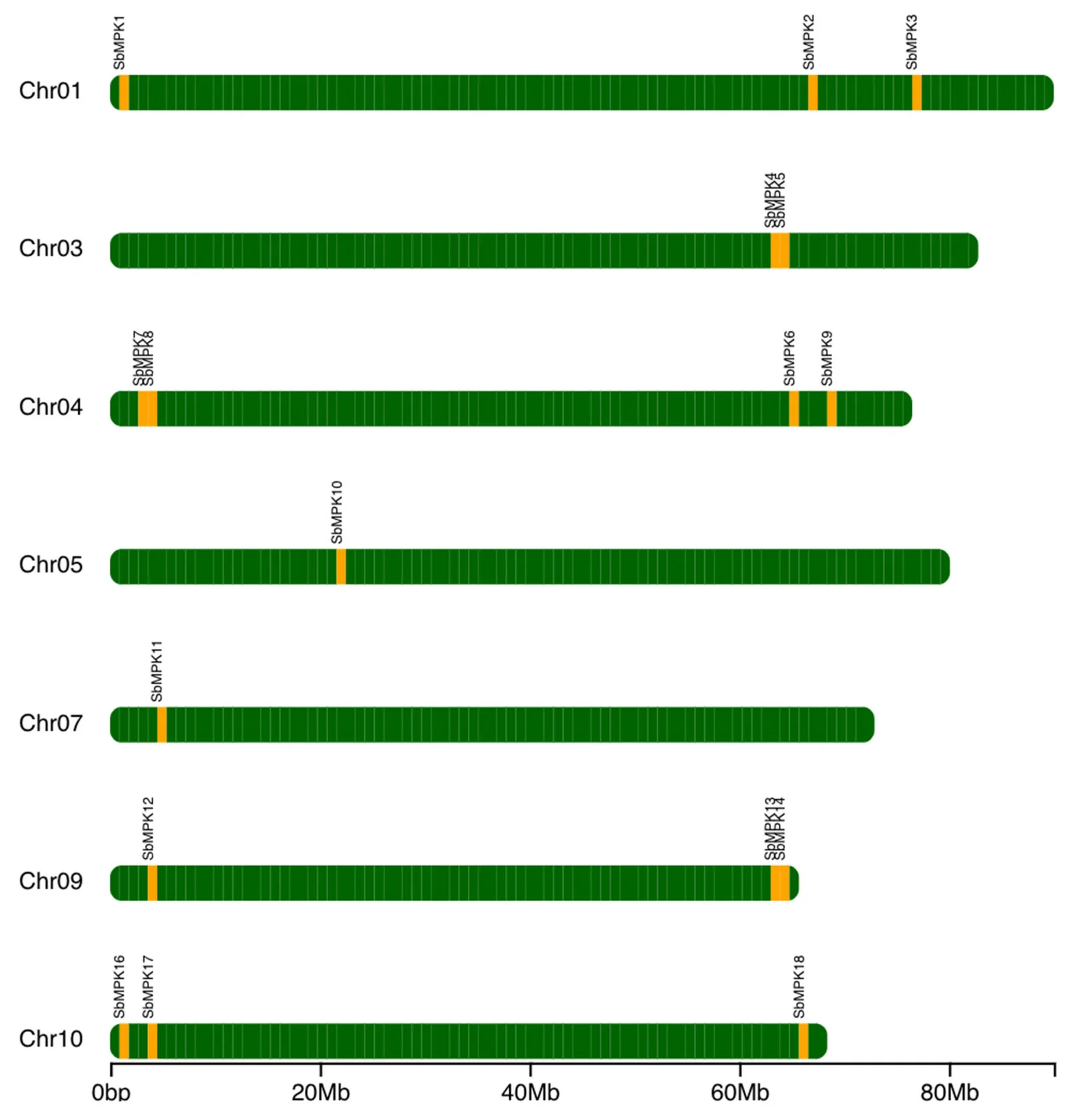
Chromosomal locations of the 19 *SbMAPK* genes in the *S. bicolor* genome. The genes are distributed on seven of the ten sorghum chromosomes. The scale at the bottom indicates chromosome length in megabases (Mb). Here, green color indicates chromosomes, and orange color specifies the gene location in the chromosome.

**Figure 5:**
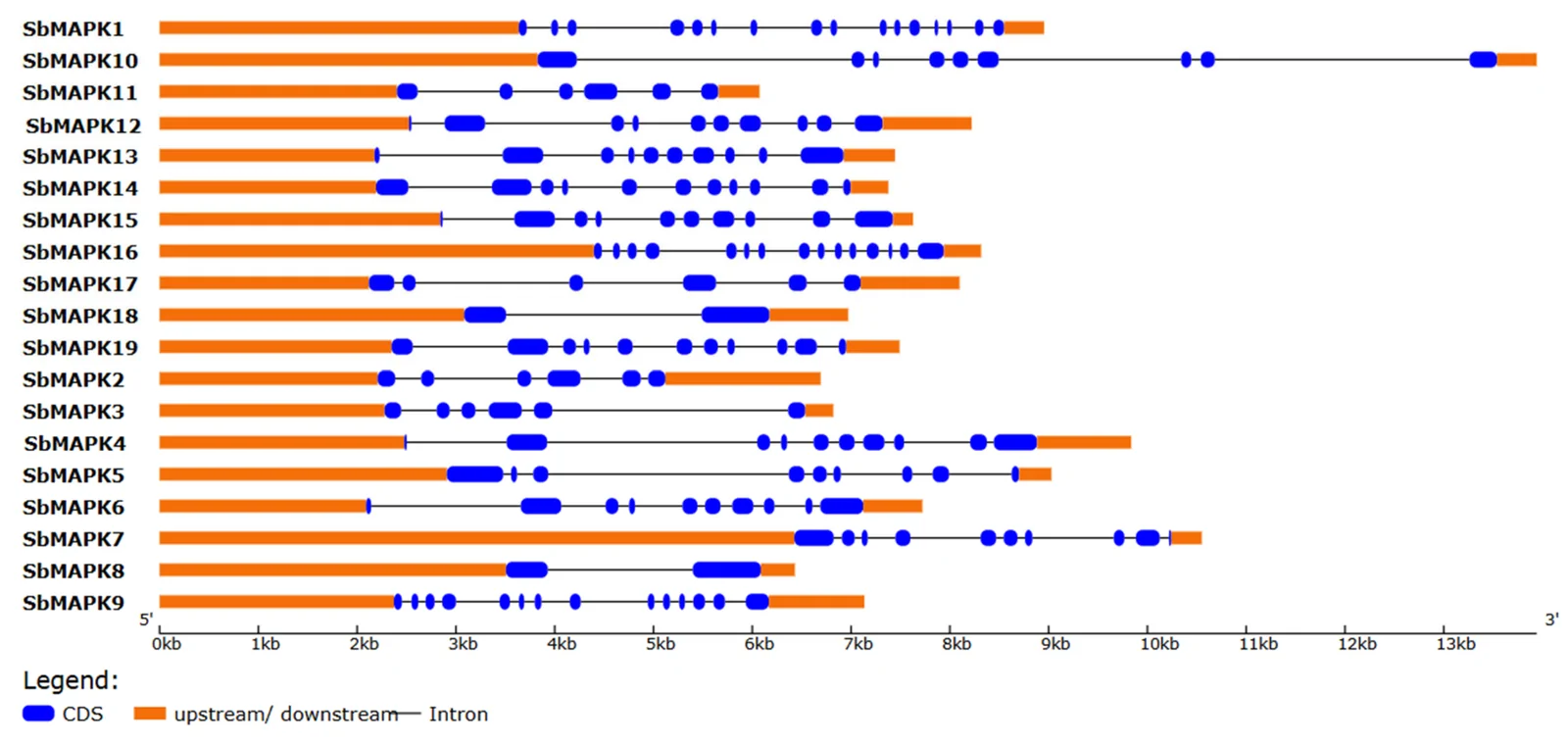
Exon–intron structures of the 19 *SbMAPK* genes. Gene structures were drawn with the Gene Structure Display Server (GSDS) using the representative gene model of each gene. Colored boxes represent coding sequences (CDSs), colored boxes represent untranslated regions (UTRs), and lines represent introns. The lengths of CDSs, introns and UTRs are drawn to scale.

These 19 *SbMAPK* genes were mapped across seven of the ten sorghum chromosomes (except Chr 2, 6 and 8), with three on Chr 1, two on Chr 3, four on Chr 4, three on Chr 9, three on Chr 10 and one on each of Chr 5 and 7 (Figure 4). All sorghum MAPKs contain 5′ and 3′ untranslated regions (UTRs) and exhibit exon numbers ranging from 2 to 16 and intron numbers ranging from 1 to 5 (Figure 5). The variations in gene structure further support the clustering of *SbMAPK* proteins in the phylogenetic tree (Figure 1).

### 2.4. Gene Duplication Analysis

Nineteen *SbMAPK* genes are displayed in the phylogenetic tree, of which there are no significant sequence similarities (i.e., more than 90%). However, based on their chromosomal locations, five gene pairs were identified as tandem duplications on chromosomes 3, 4, 9 and 10 (Table 2). These pairs are *SbMAPK4*-*SbMAPK5*, *SbMAPK7*-*SbMAPK8*, *SbMAPK13*-*SbMAPK14*, *SbMAPK16*-*SbMAPK17* and *SbMAPK6*-*SbMAPK9*. The codon-based Z test specifies nonsynonymous (dN) to synonymous substitutions (dS) in codon sequences and is used to validate codon sequence duplication by determining the dN/dS ratio. MEGA 11 software was used to determine codon sequence duplication using the codon-based Z test and dN/dS ratio. Neutral selection is shown by a dN/dS ratio of 1, positive selection is marked by a ratio > 1 and purifying selection is expressed by a ratio < 1. According to Table 2, the dN/dS ratios in each of the five gene pairs were less than 1, and the *p*-value indicated that five gene pairs had significant purifying selection, as their ratio is less than 1.

### 2.5. Cis-Element, Homology Modeling and Synteny Analysis of Sorghum MAPKs

**Figure 6:**
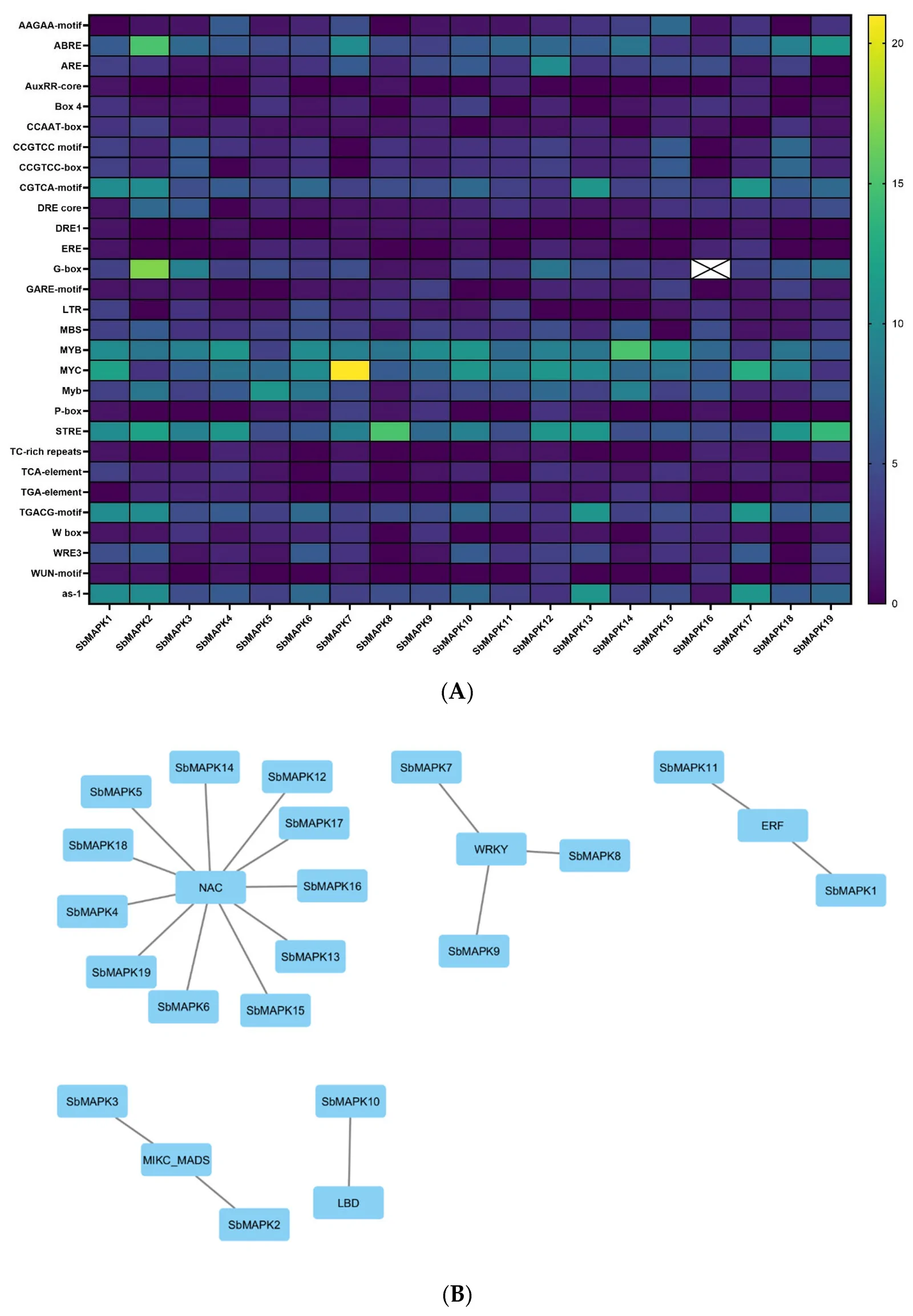
(**A**) The heatmap represents the distribution of cis-elements in the promoter regions (2000 bp upstream) of sorghum MAPK genes. The scale (color key on the right) represents the number of cis-elements in each *SbMAPK* gene. (**B**) The putative transcription factor regulatory network of the *SbMAPK* genes. The network was constructed using the PTRM tool and Cystoscope (string).

To find out the transcriptional regulation, *SbMAPK* genes were analyzed using the PlantCARE database. Distribution of the cis-elements was visualized in a heatmap (Figure 6A). All 19 *SbMAPKs* contain hormone-responsive elements, including abscisic acid (ABA) response elements (ABRE). Additionally, 11 genes have TGA elements, and 5 genes have AuxRR core (auxin response elements). It is also observed that all *SbMAPK* genes contain the Jasmonic acid (JA) response elements (TGACG-motif and CGTXA-motif) in their promoter regions. Other notable elements include the GARE-motif in 14 genes and a P-box in 9 genes, which represent gibberellin response elements. The ABA- and JA-sensitive motifs were prominent in all the *SbMAPK* genes.

Promoter analysis revealed that all *SbMAPK* cascade genes contain the cis-elements associated with both biotic and abiotic stress responses. Among the biotic-responsive elements, most of the *SbMAPKs* harbor WRE3 (expect *SbMAPK8* and *SbMAPK18*), W-box (expect *SbMAPK8*, *SbMAPK10*, *SbMAPK11*, *SbMAPK14*, and *SbMAPK18*), WUN (present in *SbMAPK1*, *2*, *4*, *7*, *12*, *16*, and *19*), and CCAAT-box (absent in *SbMAPK10*, *SbMAPK14*, and *SbMAPK17*). All 19 *SbMAPK* genes contain STRE and MBS (except *SbMAPK15*). MBS is involved in drought stress. Additionally, 18 *SbMAPKs*, all except *SbMAPK19*, possess antioxidant response elements (ARE), which are key cis-acting elements for anaerobic induction. Low-temperature response elements (LTR) were identified in 15 *SbMAPK* genes (Supplementary Table S2). These results suggested that *SbMAPK* genes play crucial roles in hormone signaling pathways and in responses to both biotic and abiotic stresses.

To further explore regulatory mechanisms, 2000 bp upstream promoter sequences of the 19 *SbMAPK* genes were analyzed using the PTRM platform to predict potential transcriptional networks. This analysis identified six transcription factors (TFs) that participated in *SbMAPK* gene regulation, which were used to construct a TF regulatory network (Figure 6B). The TF network shows that the *SbMAPK* genes are possibly regulated by the five transcription factor families, ERF, MIKC_MADS, NAC, WRKY and LBD. Among those, NAC family members were the most prevalent, followed by WRKY, ERF, MIKC_MADS and LBD.

**Figure 7:**
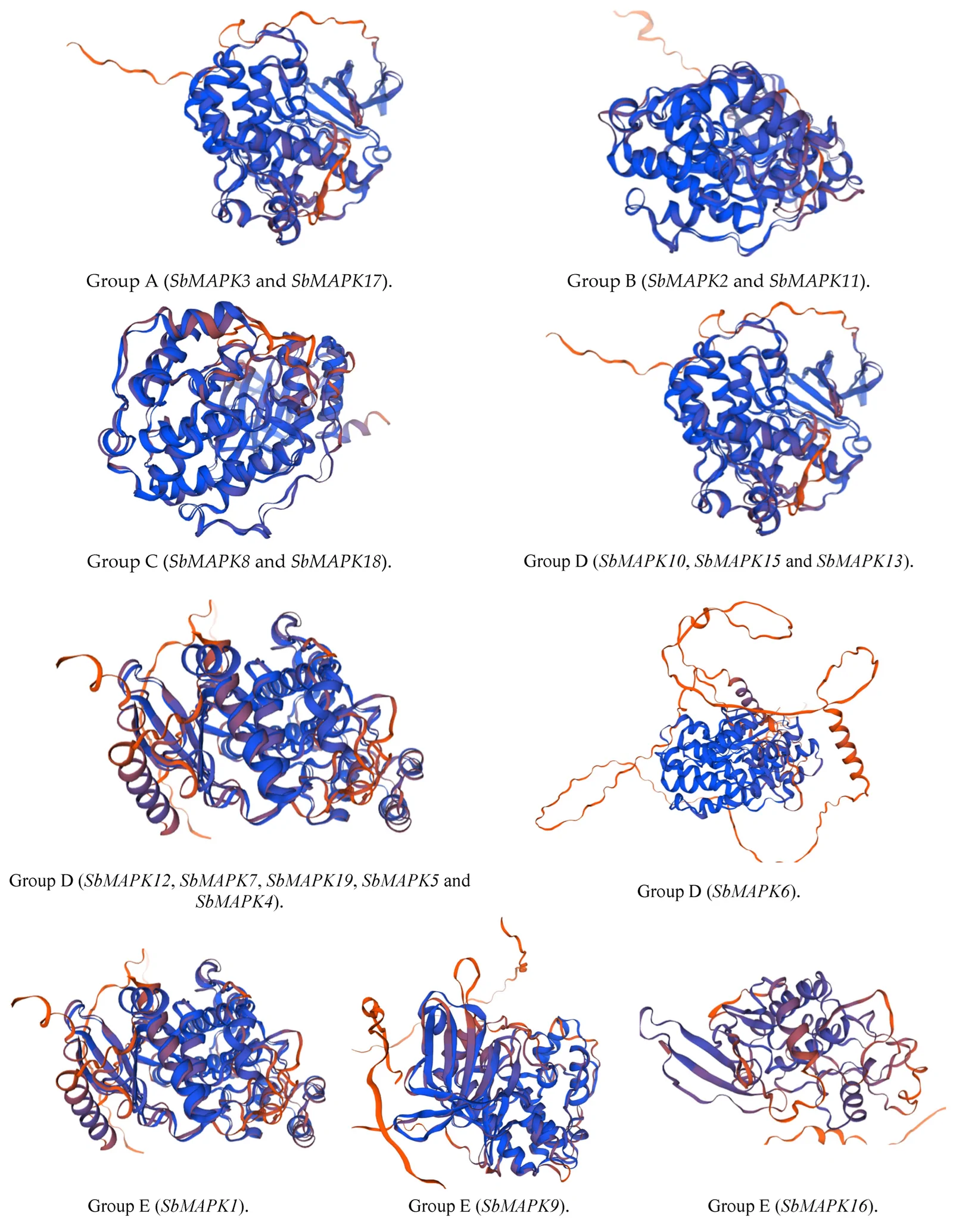
Predicted three-dimensional structures of the *SbMAPK* proteins. Models were generated by homology modeling with SWISS-MODEL. The templates and model quality scores (GMQE and QMEANDisCo) of each model are listed in Table S3.

Similar protein structures were found within the members of group A (*SbMAPK3* and *SbMAPK17*), group B (*SbMAPK2* and *SbMAPK11*) and group C (*SbMAPK8* and *SbMAPK18*) from the protein structure analysis. Group D also showed four different protein structures (i. *SbMAPK10* and *SbMAPK15*; ii. *SbMAPK12*, *SbMAPK7*, *SbMAPK19*, *SbMAPK5*, *SbMAPK4* and *SbMAPK13*; iii. *SbMAPK14*; iv. *SbMAPK6*) (Table S3). Three members of group E showed three different structures (*SbMAPK1*, *SbMAPK9* and *SbMAPK16*). On the other hand, all *SbMAPKs* have an N-terminal methionine (M) and a protein kinase (PK) domain. The TEY-type kinases contain the CD domain. In contrast, the TDY-type kinases lack this domain but feature long Ser/Thr-rich C-terminal sequences, which suggest subfamily-specific adaptability and regulatory flexibility (Figure 7). Proteins of subfamilies A and B and *SbMAPK8* and *SbMAPK18* were modeled on MAPK templates with 59% to 89% sequence identity, whereas subfamily D models were based on templates with 45% to 50% identity. *SbMAPK9* and *SbMAPK16* were best modeled on cyclin-dependent kinase templates (Table S3).

**Figure 8:**
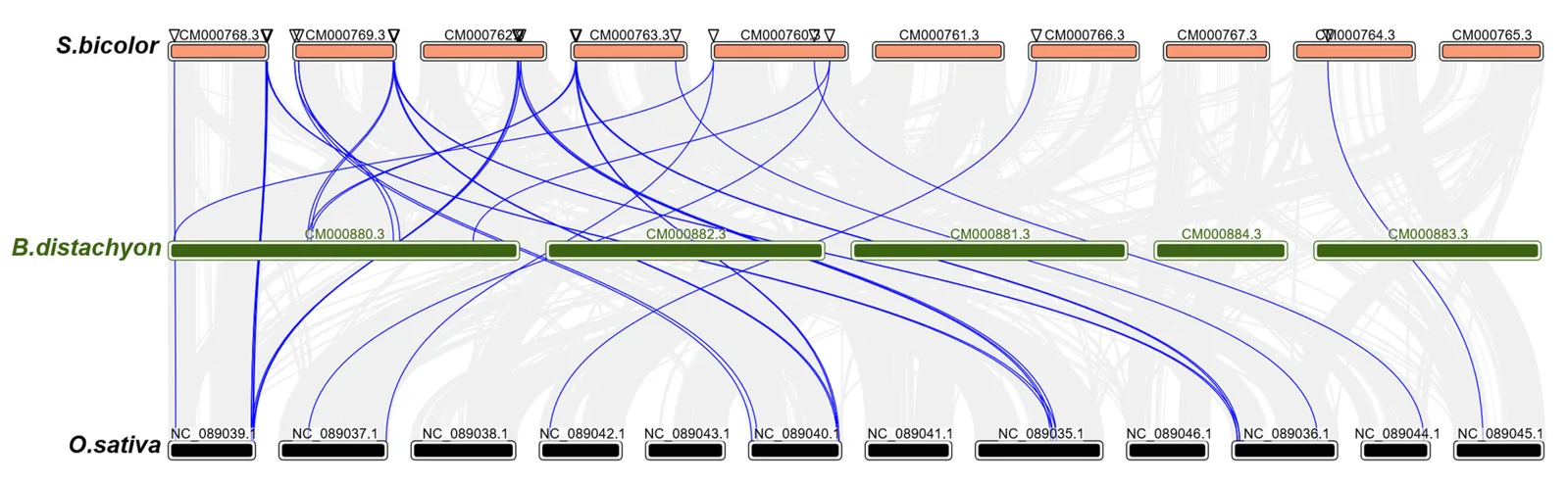
Synteny of MAPK genes between sorghum, *Brachypodium distachyon* and rice. Chromosomes of sorghum (Chr1–Chr10), *B. distachyon* (Bd1–Bd5) and rice (Chr1–Chr12) are shown as orange, green and black bars, respectively. Gray lines in the background indicate collinear blocks between the genomes, and blue lines indicate syntenic MAPK gene pairs. Triangles mark the positions of the *SbMAPK* genes on the sorghum chromosomes; closely spaced tandem genes overlap at this scale, and their exact positions are shown in Figure 4.

A synteny analysis of interspecies collinearity between sorghum and two other species (*O. sativa* and *B. distachyon*) was carried out to determine the evolutionary origin of the MAPK genes (*O. sativa* and *B. distachyon*) (Figure 8). The gray lines display collinear blocks of other chromosomes, whereas the blue line represents the collinearity of 19 *SbMAPK* genes with the two other species. More *SbMAPK* genes were found closely related to *O. sativa* than *B. distachyon*. Because sorghum (Panicoideae) is approximately equally related to rice (Oryzoideae) and *B. distachyon* (Pooideae), this difference more likely reflects genome structure: rice has retained a chromosome organization close to that of the ancestral grass genome, whereas chromosome fusions reduced the *B. distachyon* genome to five chromosomes.

### 2.6. Expression Analysis of SbMAPK Genes During Aphid Infestation

**Figure 9:**
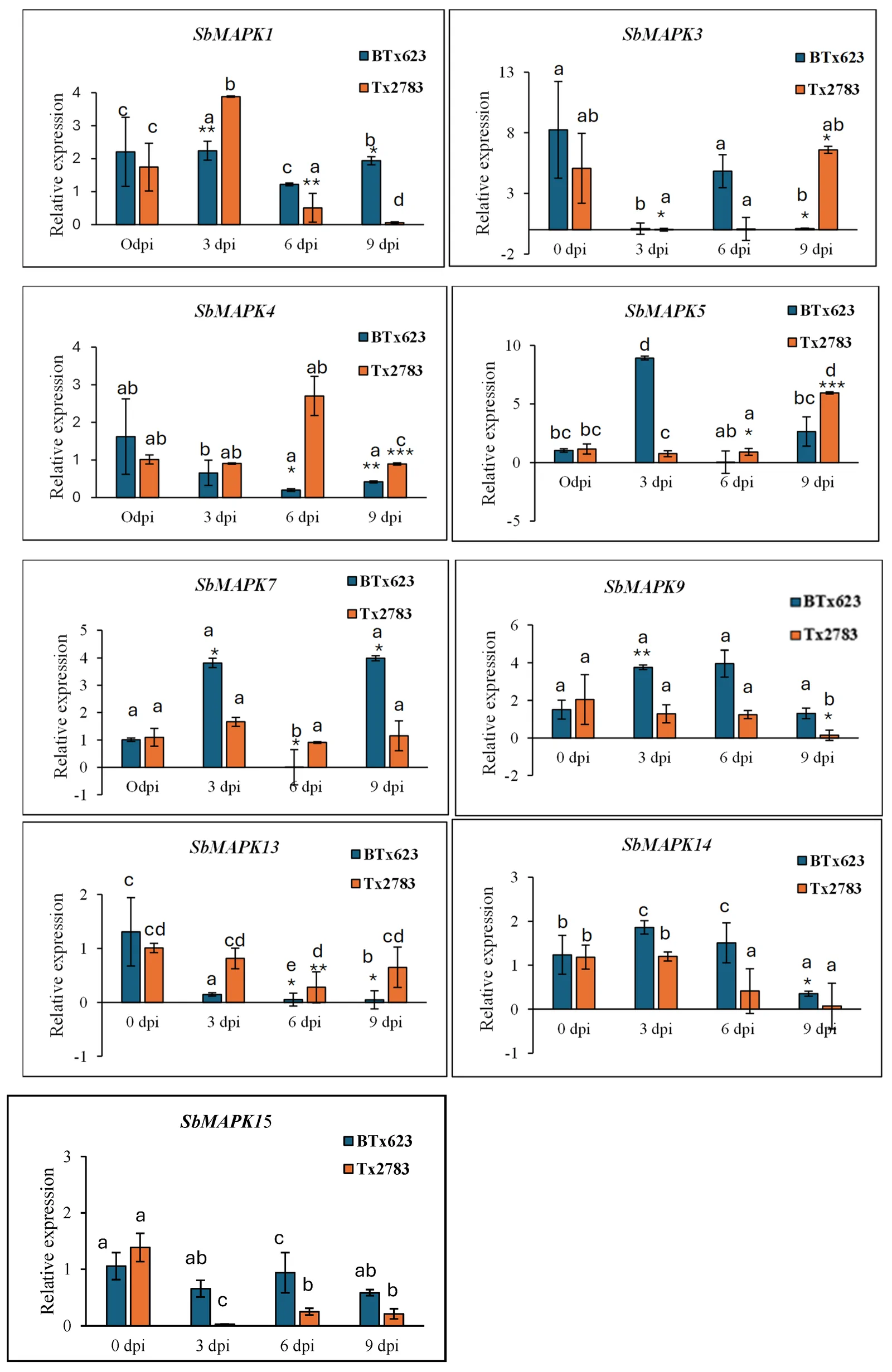
Relative expressions of nine *SbMAPK* genes in the aphid-resistant genotype Tx2783 and the susceptible genotype BTx623 during sorghum aphid infestation. Expression was measured by qRT-PCR at 0, 3, 6 and 9 days post-infestation (dpi) and calculated with the 2^−ΔΔCt^ method, using α-Tubulin as the reference gene. Bars show the mean ± standard error (*n* = 3). Different lowercase letters indicate significant differences among the genotype and time-point combinations for each gene (one-way ANOVA followed by Tukey’s HSD test, *p* < 0.05). Asterisks indicate significant differences between the control and aphid-infested samples: * *p* < 0.05, ** *p* < 0.01, *** *p* < 0.001.

Of the nine genes, seven *SbMAPKs* showed higher expression in the resistant genotype (Tx2783), except *SbMAPK13* and *SbMAPK14*. *SbMAPK3* and *SbMAPK5* showed approximately sevenfold upregulation at 9 dpi after sorghum aphid infestation. *SbMAPK1* showed an early response, while the remaining eight *SbMAPK* genes exhibited delayed responses to aphid attacks. The varying patterns of up- and down-regulation across the time points reflect dynamic interactions between the plant and aphid, indicating ongoing regulatory adjustments during aphid infestation (Figure 9).

## 3. Discussion

Plants are frequently exposed to biotic and abiotic stresses that can cause significant yield loss [19]. As sessile organisms, plants cannot escape unfavorable environmental conditions and have therefore evolved complex defense and signaling pathways to cope with diverse stresses. Among these pathways, mitogen-activated protein kinase (MAPK) cascades play essential roles in regulating plant growth, development and responses to environmental challenges [20,21,22]. Because MAPK genes are widely distributed across terrestrial plants, including eudicots and mosses, characterizing their sequences, structures and evolutionary relationships can provide insights into their functional diversification and evolutionary history [23]. MAPK gene families have been extensively studied in several species, including Arabidopsis, rice, maize and cotton [6,8,24,25]. However, the sorghum MAPK gene family remains comparatively underexplored. In this study, we conducted a genome-wide analysis of the sorghum MAPK (*SbMAPK*) gene family, examining its phylogenetic relationships, gene structures, evolutionary patterns, predicted regulatory elements, protein structures and expression responses to sorghum aphid infestation.

Multiple sequence alignments confirmed that all 19 sorghum *SbMAPK* genes possess conserved MAPK signature motifs, including the canonical TXY motifs. The number of TXY motifs in sorghum is consistent with those in *A. thaliana* [9,10], *B. distachyon* [14], *O. sativa* [11], *T. aestivum* [12], *C. sinensis* [15] and *P. trichocarpa* [16]. All 19 MAPKs exhibit basic isoelectric points (PI) ranging from 5.31 to 9.66, comparable to values observed in *L. sativa* (4.74 to 9.08) [26] and *F. ananassa* (5.05 to 9.34) [27]. Gene structure analysis revealed exon numbers ranging from 2 to 16 and intron numbers from 1 to 5, like *H. annuus* (1–17) [28]. Notably, members within subfamily A (*SbMAPK3* and *SbMAPK17*), subfamily B (*SbMAPK2* and *SbMAPK11*) and subfamily C (*SbMAPK1*, *SbMAPK8*, *SbMAPK9*, *SbMAPK16* and *SbMAPK18*) shared similar exon–intron architectures, while members in subfamily D showed more variation, mirroring structural diversity found in strawberry [27]. Subfamily A contained TEY motifs, C comprised both TEY and TDY motifs, D possessed only TDY motifs and B featured both TEY and MEY motifs. Similar group B findings were found in *B. distachyon* (*BdMAPK11*) and tomato, although tomato is a dicot [14,29]. Collectively, these conserved and variable features provide a basis for understanding the structural organization and potential functional diversification of *SbMAPKs*.

To investigate evolutionary relationships between sorghum MAPK proteins, an unrooted maximum likelihood phylogenetic tree was created using 128 MAPK amino acid sequences from six plant species, including monocots (*Z. mays*, *Setaria italica* and *B. distachyon*), eudicot (*A. thaliana*), angiosperms (*P. trichocarpa*) and *S. bicolor*. With these MAPKs, *Chlamydomonas reinhardtii cyclin-dependent kinases* (*CrCDK*) [30], Saccharomyces cerevisiae Fus3 protein (Fus3) [31] and Homo sapiens cyclin-dependent kinases 1 and 17 (*HsCDK1 and HsCDK17*) [32,33] were the outgroup. The 19 *SbMAPKs* were clustered into five subfamilies (subfamilies A, B, C, D and E) in line with the previous classifications in soybean [34] and kiwifruit MAPKs [35]. While the rice MAPK family has been divided into six subfamilies [4,36] and the kiwifruit MAPKs into five [35], such discrepancies likely result from methodological differences in tree construction. In this study, subfamilies A, B and C contained 2 *SbMAPK* genes each, and the subfamily D contained 11 *SbMAPK*, which is the largest and most diverse cluster, and is consistent with observations in rice, tomato, poplar and *Arabidopsis* [9,24,29,37]. The expansion of subfamily D suggests that its members may have undergone lineage-specific diversification, although additional functional and evolutionary analyses are needed to determine whether this expansion is associated with distinct biological roles.

Gene duplication is a major driver of plant genome evolution, contributing to gene family expansion, genetic diversity and the emergence of novel regulatory networks. Tandem and segmental duplications are the two major mechanisms underlying the expansion of gene families in plant genomes [38,39]. Following duplication, genes may evolve under purifying, neutral or positive selection. In the present study, analyses based on dN/dS ratios and codon-based Z tests indicated that the *SbMAPK* gene family has evolved predominantly under neutral and purifying selection. All five identified duplicated gene pairs exhibited dN/dS ratios below 1, suggesting strong purifying selection (Table 2). Segmental duplication was identified as the predominant mechanism contributing to the expansion of the *SbMAPK* gene family. Similar patterns of purifying selection have been reported for MAPK gene families in other plant species, including *Arabidopsis thaliana*, *Oryza sativa* and *B. distachyon* [40]. Following duplication, MAPK genes may retain ancestral functions, thereby contributing to plant responses to diverse biotic and abiotic stresses and supporting broad environmental adaptability. Such functional conservation has also been reported in cotton (*Gossypium raimondii*) and other crop species [25,41]. Nevertheless, the specific contributions of individual duplicated *SbMAPKs* to stress responses remain to be experimentally determined.

Cis-regulatory elements play important roles in controlling gene expression by mediating interactions between transcriptional factors and gene promoter regions, thereby contributing to the regulation of protein-coding genes and noncoding RNAs (lncRNAs) [42,43]. In this study, promoter analysis identified diverse predicted cis-regulatory elements associated with phytohormone signaling and responses to environmental stresses in *SbMAPK* genes. These included hormone-responsive elements associated with jasmonic acid (JA), gibberellin (GA), abscisic acid (ABA) and auxin, as well as elements associated with abiotic stress responses, such as STRE, MBS, DRE1, LTR and ARE, and biotic stress responses, including WRE3, W-box, WUN-motif, CCAAT-box and TC-rich repeats (Table S2). MYB-binding elements were particularly abundant in the *SbMAPK* promoters and have been associated with enhanced expression in the resistant genotype Tx2783 as well as circadian regulation in sorghum [44,45]. These findings are consistent with previous studies in strawberry [27], cotton [41] and kiwifruit [35], in which MeJA- and ABA-responsive elements were frequently identified in MAPK promoters. Furthermore, OsMAPK5 in rice has been reported to be ABA-inducible and to contribute to both disease resistance and tolerance to abiotic stresses [44]. The presence of similar hormone- and stress-responsive cis-regulatory elements in *SbMAPK* promoters suggests that these genes may be responsive to hormonal and environmental signals. However, experimental analyses are required to confirm the functionality of these elements and determine their contributions to *SbMAPK* expression.

Transcription factors (TFs) are central regulators of gene expression and coordinate diverse biological processes, including plant development, responses to environmental stimuli, and activation of defense mechanisms [46,47]. Identifying and characterizing potential TF-associated regulatory networks can therefore help elucidate the mechanisms underlying *SbMAPK* regulation [46]. In this study, five major TF families—ERF, MIKC_MADS, NAC, WRKY and LBD—were identified in the predicted promoter regions associated with the sorghum MAPK genes (Figure 5), with NAC representing the most abundant family. NAC TFs regulate plant defense through both ABA-dependent and ABA-independent pathways and contribute to responses to wounding, pathogen infection, and insect infestation in Arabidopsis and rice [48,49]. NAC TFs have also been shown to be induced in sorghum following greenbug aphid infestation, where they participate in regulatory networks associated with genetic resistance [50]. Similarly, ERF TFs have important roles in plant responses to both biotic and abiotic stresses [42]. For example, ERF genes in maize have been associated with resistance to *Exserohilum turcicum* [51], whereas an ERF gene from Amur grape enhanced resistance to *Pseudomonas syringae* and *Botrytis cinerea* [52]. The WRKY, MIKC_MADS and LBD families are also involved in plant development and stress responses. MIKC_MADS TFs regulate processes such as root, flower and seed development, whereas LBD gene expression in soybean is responsive to hormonal signals as well as cold, drought and salinity stresses [53]. MAPK signaling is one of the earliest defense responses activated following herbivore attack [54]. Key components of this response include MAPKs, WRKY TFs, calmodulin (CaM/CALM) and respiratory burst oxidase homolog (RBOH) proteins [44]. Collectively, the abundance of these TF-associated cis-regulatory elements in *SbMAPK* promoter regions suggests that *SbMAPK* genes may be regulated by diverse transcriptional networks involved in hormonal signaling and responses to biotic and abiotic stresses in sorghum. Nevertheless, experimental validation is needed to establish direct TF–promoter interactions and determine how these regulatory relationships influence *SbMAPK* expression during aphid infestation.

Three-dimensional protein structural analysis further supported the conservation of the N-terminal protein kinase (PK) domain across all *SbMAPK* proteins. In contrast, variation in the activation-loop motifs, particularly the TDY, TEY and MEY motifs, distinguishes the different *SbMAPK* subfamilies and may reflect their evolutionary divergence and functional specialization. These structural differences provide potential clues to functional diversity among *SbMAPKs*, although their effects on kinase activity and substrate specificity remain to be investigated experimentally.

Following herbivore infestation, MAPK signaling can rapidly activate and modulate phytohormone biosynthesis and signaling, leading to changes in the transcriptome and proteome that contribute to plant defense responses [55]. In the present study, nine of the 19 *SbMAPK* genes exhibited differential expression following aphid infestation. Four genes (*SbMAPK3*, *SbMAPK5*, *SbMAPK7* and *SbMAPK13*) were upregulated in the resistant genotype Tx2783, whereas five genes (*SbMAPK1*, *SbMAPK4*, *SbMAPK7*, *SbMAPK9* and *SbMAPK14*) were upregulated in the susceptible genotype BTx623. *SbMAPK7* was responsive in both genotypes, suggesting that it may participate in general response to aphid infestation rather than a resistance-specific response (Figure 9). However, differences in expression magnitude and timing between the two genotypes may also reflect distinct regulatory responses. These expression patterns identify candidate *SbMAPKs* for further investigations but do not, by themselves, establish a direct role in aphid resistance.

Among the identified *SbMAPKs*, *SbMAPK3* and *SbMAPK17* are orthologous to the well-characterized Arabidopsis thaliana MAPKs *AtMAPK3* and *AtMAPK6*, respectively, and belong to subfamily A. These MAPKs participate in responses to a broad range of environmental and hormonal signals [56]. *AtMAPK3* is induced by cold, heat, salt, 1-aminocyclopropane-1-carboxylic acid (ACC), salicylic acid (SA) and JA, suggesting that its sorghum ortholog may similarly function at the intersection of abiotic stress and hormone signaling pathways. *SbMAPK2* and *SbMAPK11* belong to subfamily B and are phylogenetically related to Arabidopsis subfamily B MAPKs, including *AtMAPK4*, *AtMAPK5*, *AtMAPK11*, *AtMAPK12* and *AtMAPK13* (Figure 1). Members of this group have been involved in diverse biological processes, including cell division, plant immunity and adaptation to environmental stresses [57,58]. Sorghum subfamily C comprises *SbMAPK1*, *SbMAPK8*, *SbMAPK9*, *SbMAPK16* and *SbMAPK18*. In Arabidopsis, *AtMAPK1* has been implicated in responses to salt stress [59], while *AtMAPK1* and *AtMAPK2* can be activated by ABA [60]. Subfamily D MAPKs are also associated with diverse stress-response and defense-related processes [61], including regulation of microtubule organization and stomatal movement [62]. These comparative findings provide a framework for generating hypotheses about *SbMAPK* function, but function conservation between sorghum and other plant species requires experimental verification.

This study represents a comprehensive investigation of the MAPK gene family in sorghum, including the identification and characterization of all family members and the first evaluation of their expression responses to sorghum aphid infestation. Our results identified several *SbMAPKs* potentially involved in plant–aphid interactions, providing a foundation for further investigation of their roles in host defense. However, the current evidence is correlative, as our analysis focused on gene expression patterns following aphid infestation. Functional studies, including gene silencing, gene editing or overexpression combined with aphid performance assays, are needed to determine whether these genes directly contribute to aphid resistance. Similarly, the cis-regulatory element, transcription factor and protein structural analyses were based on computational predictions that require experimental validation. These analyses do not establish transcription factor binding, promoter activity or co-expression relationships, and some protein homology models were based on templates with limited sequence similarity. In addition, alternative splicing and tissue-specific expression were not investigated.

Our results revealed genotype- and time-dependent expression responses among *SbMAPKs* following aphid infestation. *SbMAPK3*, *SbMAPK4* and *SbMAPK5* showed increased expression at later time points in the resistant genotype, with *SbMAPK4* exhibiting significantly higher expression at 6 and 9 days post-infestation (DPI). These findings identify *SbMAPK4* as a promising candidate for further investigation of its potential role in aphid resistance. In contrast, the early induction of some *SbMAPKs* in the susceptible genotype may reflect responses associated with aphid pressure or basal defensive response rather than action of specific host resistance to aphids.

## 4. Materials and Methods

### 4.1. Identification of MAPKs in the Sorghum Genome

To identify the MAPK gene family in sorghum, two complementary search strategies were used. First, the MAPK protein sequences of *Brachypodium distachyon* were downloaded from the Phytozome database (https://phytozome-next.jgi.doe.gov/, 10 January 2023) and used as queries for blastp against the *Sorghum bicolor* (L.) genome (version v3.1.1) in the Phytozome collection, with an E-value of 1 × 10^−10^ as the threshold and alignment number of 500. Second, the hidden Markov model (HMM) profile of the protein kinase domain, PF00069, was obtained from the Pfam database and used to search the proteome with HMMER. From the obtained sequences, redundant sequences and alternative transcripts of the same locus were removed, keeping the representative gene of each locus. Because PF00069 is shared by all kinases, candidate MAPKs were identified if they contain the MAPK signature sequence TXYxxTRWYRAPE in the multiple sequence alignment with known *B. distachyon* MAPK [14]. Further, these potential MAPK proteins were checked using the InterPro program to confirm the MAPK domain, which is PF00069 [10]. Following the guidelines for Arabidopsis MAPK nomenclature, each gene was assigned a two-letter code that matched Sorghum bicolor (Sb) and then the family designation (MAPK) [9].

### 4.2. Physicochemical Properties and Subcellular Localization

To characterize the identified proteins, the theoretical isoelectric point (PI) and molecular weight (MW) of each sorghum MAPK were calculated using the Compute PI/Mw tool in the ExPASy database (http://www.expasy.org/), and subcellular localizations were identified using the WoLF PSORT online program (https://wolfpsort.hgc.jp/ (accessed on 21 October 2026)).

### 4.3. Sequence Acquisition and Phylogenetic Analysis of Sorghum MAPKs

From the databases of the National Center for Biotechnology Information (NCBI) (http://www.ncbi.nlm.nih.gov/) and Phytozome, MAPK protein sequences were retrieved from five different plant species, including monocot (*Z. mays*, *Setaria italica* and *B. distachyon*) and eudicot (*A. thaliana* and *P. trichocarpa*); for the outgroup: *Chlamydomonas reinhardtii* cyclin-dependent kinases (*CrCDK*), *Saccharomyces cerevisiae Fus3* protein (*Fus3*) and *Homo sapiens cyclin-dependent kinases 1 and 17* (*HsCDK1* and *HsCDK17*). Their gene IDs are listed in Tables S4–S6. Then, multiple sequence alignments were performed among these collected protein sequences using Clustal Omega with its default parameters (https://www.ebi.ac.uk/jdispatcher/msa/clustalo, version 2.0, (accessed on 21 October 2026)) [63]. A maximum likelihood phylogenetic tree was constructed based on MAPK protein sequences using MEGA 11 software with the Jones–Taylor–Thornton (JTT) model using bootstrap of 1000 replications to establish evolutionary relationships among these MAPK genes [64,65].

### 4.4. Gene Structure, Chromosomal Location and Conserved Motifs

To compare gene organization among *SbMAPKs*, exon–intron structures were displayed using the Gene Structure Display Server (GSDS) (https://gsds.gao-lab.org/) based on the genomic and coding sequences of the representative gene models. The number of annotated transcript isoforms of each gene was obtained from the Phytozome annotation. The chromosomal positions of the *SbMAPK* genes obtained from Phytozome were used to create the chromosome map with the chromoMap package in R [66].

To identify conserved protein motifs, the *SbMAPK* protein sequences were analyzed with the MEME Suite (https://meme-suite.org/meme/ (accessed on 21 October 2026)), with the maximum number of motifs set at 10. Sequence logos of the conserved activation-loop region were generated using WebLogo 3 [67]. The presence of a complete common docking (CD) domain and the length of the C-terminal region beyond the kinase domain were determined from the multiple sequence alignment for each *SbMAPK*.

### 4.5. Determination of Gene Duplication and Calculation of dN/dS Ratio

From the genome mapping, the closely located gene pairs were selected for gene duplication analysis. Coding sequences of the *SbMAPK* genes were used to calculate the synonymous (dS) and nonsynonymous (dN) rates of substitution using MEGA 11 according to Han and Luthe [68]. These sequences were first aligned in MEGA 11 software using Clustal W after the dN and dS were estimated using Jones–Taylor–Thornton (JTT) substitution model [69]. Finally, to get a *p*-value of the selection, a codon-based Z-test was performed on each pair of DNA sequences. With the *p*-value (<0.05 is considered significant at the 5% level) of each pair of sequences, neutral evolution (dN = dS), positive selection (dN > dS) or purifying selection (dN < dS) were tested on each pair of DNA sequences [70].

### 4.6. Analysis of SbMAPK Promoters, Prediction of Cis-Regulatory Elements and Three-Dimensional Protein Structure

The promoter sequences of approximately 2000 base pairs upstream of all *SbMAPK* genes were retrieved from Phytozome and were analyzed using the PlantCARE promoter analysis tool [71]. Following the identification of various cis-elements in the promoter region, a heatmap was generated using the SRplot website [72]. The Plant Transcriptional Regulatory Map (PTRM) (http://planttfdb.gao-lab.org/prediction.php (accessed on 21 October 2026)) was used with the promoter sequence to predict the transcription factors. Cytoscape 3.8.2 was used to visualize the transcription factor regulatory network with these transcription factors [73]. The three-dimensional structure of the *SbMAPK* proteins was analyzed using the SWISS-MODEL online server (https://swissmodel.expasy.org/) for homology-based modeling and structural prediction.

### 4.7. Synteny Analysis

Synteny analysis was conducted by comparing the genomic locations of *SbMAPK* genes to those of closely related species like *O. sativa* and *B. distachyon* in our study. We utilized Tbtools to analyze the species’ gene order conservation as well as to view the synteny maps.

### 4.8. Plant Growth and Aphid Infestation

Two sorghum genotypes, Tx2783 (resistant) and BTx623 (susceptible), were selected for this study. Seeds of these genotypes were sown in plastic pots containing potting compost (Sungro Professional Growing Mix, Agawam, MA, USA) after being treated with fungicide (Captan 50W, Bonham, TX, USA). The plants were grown in a greenhouse set at a temperature of (28 ± 2) and 60% relative humidity under a constant photoperiod of 14 h-light/10 h-dark. The sorghum aphid colonies were cultured on a susceptible sorghum line Tx7000. Ten- to twelve-day-old (2–3 leaf stage) seedlings were infested with 20 adult apterous aphids on the adaxial side of the first true leaf. The aphid-infested and control plants were kept in transparent cages with nylon mesh on the top to avoid sorghum aphid escape and unwanted pathogen/pest infestation. To evaluate the differential responses of the two genotypes to aphid infestation, changes in aphid numbers during the co-culture were recorded at 0, 3, 6, and 9 days post-infestation (dpi) from ten individual plants of each infested genotype among three replications. At the same time, all seedlings were rated for their responses to aphid feeding. For sample collection, aphids were removed from the plants at each of the time points, and each treatment had three biological replicates, which were harvested and frozen in liquid nitrogen, then stored at −80 °C for future use.

### 4.9. RNA Extraction and qRT-PCR Analysis

The seedling tissues from aphid-infested and control plants were ground into a fine powder in liquid nitrogen and mixed with Trizol reagent (Invitrogen, Carlsbad, CA, USA) (according to the manufacturer’s protocol) to extract total RNA from each sample. Then the RNA samples were treated with DNase (Turbo DNA-free kit, Thermal Fisher Scientific, Waltham, MA, USA) to remove any DNA in the RNA solution. To confirm the quality and integrity of the extracted RNAs, Nanodrop lite spectrophotometer (Thermo Fisher Scientific, Wilmington, DE, USA) and agarose gel electrophoresis were used. For real-time quantitative PCR (qRT-PCR) analysis, the purified RNA was converted to cDNA using the GoScript reverse transcriptase kit (Promega, Madison, WI, USA) following the manufacturer’s instructions. The resulting cDNAs were diluted in a 1:4 ratio for qRT-PCR analysis to verify the differentially expressed MAPK genes that were identified from our previous RNA-seq data, which was conducted with the same sorghum lines and sorghum aphid [44]. The primers were designed using the IDT DNA program (https://www.idtdna.com/PrimerQuest/Home/Index, 15 June 2023) (Coralville, Johnson County, IA, USA) and are listed in Table S7. The qRT-PCR experiments were performed with three biological and two technical replicates for each sample on a Bio-Rad iCycler thermal cycler (Bio-Rad Laboratories, Inc., Hercules, CA, USA) using the iTaq™ universal SYBR^®^ green supermix (Bio-Rad Laboratories, Inc.). A housekeeping gene in sorghum, α-Tubulin gene (Sobic.001G107200), was used as the internal control [74]. The qRT-PCR assay was conducted in a 10 μL volume, comprising 1 μL of cDNA, 0.4 μL of each reverse and forward primer (at a concentration of 10 μM each), 5 μL of SYBR green master mix and 3.2 μL of double-distilled water (ddH2O). The following conditions were used for the qRT-PCR reaction: one cycle at 95 °C for 3 m, 40 cycles at 95 °C for 10 s and 55 °C for 30 s, followed by one cycle each of one min at 95 °C and 55 °C. The final melting curve was 81 cycles at 55 °C for 30 s [70].

### 4.10. Statistical Analysis

The qRT-PCR results reported here are the averages of three biological and two technical replicates, and the relative expression level of each gene was calculated using the 2^−ΔΔCt^ method [75]. For each gene, differences among genotype and time-point combinations were tested by one-way analysis of variance (ANOVA) followed by Tukey’s honestly significant difference (HSD) test (*p* < 0.05) using R version 4.3.2.

## 5. Conclusions

In summary, we identified 19 MAPK genes and three MAPK-like genes in *Sorghum bicolor* and characterized their phylogenetic relationships, gene structures, evolutionary patterns and predicted regulatory features. Duplicated *SbMAPK* genes have been maintained under purifying selection, and their promoters contain predicted cis-regulatory elements associated with phytohormone signaling and stress responses. Following sorghum aphid infestation, nine *SbMAPKs* exhibited genotype- and time-dependent expression patterns. Notably, *SbMAPK3*, *SbMAPK4* and *SbMAPK5* were induced primarily at later stages in the resistant genotype Tx2783, whereas several other genes responded earlier in the susceptible genotype BTx623. These findings lead to identification of candidate genes for further investigation of their roles in aphid resistance. Future research should focus on (i) functionally validating these candidates through gene silencing or overexpression combined with aphid bioassays; (ii) determining whether MAPK protein activation differs between resistant and susceptible genotypes following aphid infestation; and (iii) experimentally validating the predicted cis-regulatory elements and transcriptional regulators. If their contributions to aphid resistance are confirmed, these genes could provide valuable targets for molecular marker development and genome editing to support the breeding of aphid-resistant sorghum.

## Figures and Tables

**Table 1:** Structural features of 19 MAPK genes found in *S. bicolor* L.

Gene ID	Gene Name	Chr	Genomic Sequence	Transcript Sequence	CDS	ORF (AA)	Location Coordinates	Orientation	PI	MW (kDa)	GRAVY
**Sobic.001G014200**	*SbMAPK1*	1	6954	2176	1293	431	1289674..1296628	reverse	5.31	42.89	-0.269
**Sobic.001G315700**	*SbMAPK2*	1	4692	2406	1134	378	60385343..60390035	reverse	6.07	42.64	-0.308
**Sobic.001G410100**	*SbMAPK3*	1	4820	1686	1125	275	69359088..69363908	forward	5.52	43.42	-0.301
**Sobic.003G229400**	*SbMAPK4*	3	7836	3273	1842	614	56782007..56789843	reverse	9.16	69.96	-0.54
**Sobic.003G236000**	*SbMAPK5*	3	7028	2723	1488	496	57471899..57478927	reverse	7.68	56.66	-0.454
**Sobic.003G248100**	*SbMAPK6*	3	5722	2466	1776	592	58689765..58695487	forward	9.38	67.38	-0.535
**Sobic.004G032800**	*SbMAPK7*	4	8550	5397	1473	491	2628619..2637169	reverse	8.97	56.08	-0.473
**Sobic.004G040200**	*SbMAPK8*	4	4431	2274	1113	371	3307839..3312270	reverse	6.7	42.43	-0.135
**Sobic.004G270200**	*SbMAPK9*	4	5135	2017	1371	457	61456135..61461270	reverse	9.66	51.16	-0.46
**Sobic.005G106600**	*SbMAPK10*	5	1194	3329	1623	541	20000010..20011951	forward	8.75	61.51	-0.499
**Sobic.007G046100**	*SbMAPK11*	7	4073	1986	1167	389	4636467..4640540	forward	6.25	44.05	-0.298
**Sobic.009G038900**	*SbMAPK12*	9	6221	3097	1671	557	3630509..3636730	forward	8.85	63.55	-0.559
**Sobic.009G234300**	*SbMAPK13*	9	5445	2469	1773	591	57362559..57368004	reverse	9.21	67.23	-0.522
**Sobic.009G242200**	*SbMAPK14*	9	5377	2351	1779	593	57877841..57883218	forward	6.7	66.84	-0.512
**Sobic.009G246300**	*SbMAPK15*	9	5628	2842	1797	599	58190617..58196245	reverse	9.3	67.38	-0.483
**Sobic.010G013200**	*SbMAPK16*	10	6316	2726	1422	474	1098112..1104428	forward	9.43	53.97	-0.478
**Sobic.010G043200**	*SbMAPK17*	10	6098	2329	1209	403	3339727..3345825	reverse	5.32	45.51	-0.345
**Sobic.010G249500**	*SbMAPK18*	10	4971	2111	1110	370	58902074..58907045	forward	6.86	42.4	-0.167
**Sobic.010G257100**	*SbMAPK19*	10	5491	2614	1725	575	59522250..59527741	forward	6.74	65.52	-0.516

Chr—chromosome; UTR—Untranslated regions; CDS—Coding DNA sequence; MW—Molecular weight; IP—Isoelectric point.

**Table 2:** Analysis of gene duplication of the MAPK gene pairs in sorghum.

Gene Pairs	dN	dS	dN/dS	*p*-Value
*SbMAPK4-SbMAPK5*	0.128	0.851	0.151	0.00000001 *
*SbMAPK7-SbMAPK8*	0.361	0.790	0.457	0.00000001 *
*SbMAPK13-SbMAPK14*	0.148	0.834	0.178	0.00000001 *
*SbMAPK16-SbMAPK17*	0.493	0.784	0.629	0.000000001 *
*SbMAPK6-SbMAPK9*	0.484	0.753	0.643	0.000000001 *

Here, dN = nonsynonymous substitution rate, dS = synonymous substitution rate. To be considered purifying selection, dN/dS < 1 and *p*-value for the Z-test should be below 0.05 (* *p* < 0.05).

## Data Availability

The data that supports the findings of this study are available in the supporting materials of this article, or available upon request.

## Supplementary Materials

The following supporting information can be downloaded at: https://www.mdpi.com/article/10.3390/plants15213342/s1, Table S1: Subcellular localization and UTR lengths of *SbMAPK* genes; Table S2: Cis-regulatory elements in *SbMAPK* promoters; Table S3: Homology models of *SbMAPK* proteins; Table S4: MAPK genes of sorghum, Brachypodium and Arabidopsis; Table S5: MAPK genes of maize, poplar and foxtail millet; Table S6: Outgroup sequences used in the phylogenetic analysis; Table S7: qRT-PCR primers.

## Abbreviations

MAPK	Mitogen-activated protein kinase
MAPKK	Mitogen-activated protein kinase kinase
MAPKKK	Mitogen-activated protein kinase kinase kinase
*SbMAPK*	*Sorghum Mitogen-activated protein kinase*
qRT-PCR	Quantitative real-time PCR
PI	Theoretical isoelectric point
MW	Molecular weight
NJ	Neighbor-joining
ABA	Abscisic acid
JA	Jasmonic acid
GA	Gibberellic acid
ABRE	Abscisic acid response elements
MeJA	Methyl jasmonate
TF	Transcription factor
GARE	Gibberellic Acid Response Element
ERF	Ethylene-responsive element binding factors
LTR	Low-temperature response elements
STRE	Stress Response Element
MBS	MYB-binding site
lncRNA	Long noncoding RNA
RbOHD	Respiratory burst oxidase homolog protein
