# Supplementary material for: Identification and Characterization of the MAPK Genes in Sorghum and Their Expression in Response to Aphid Infestation
Source: Plants (Basel). 2026 Oct 28;15(21):3342. doi: 10.3390/plants15213342 (PMC13765962; doi:10.3390/plants15213342)
Supplement: Supplementary file 1 [file plants-15-03342-s001.zip › plants-4565146-supplementary.pdf]

Table S1: Subcellular location and UTR position of the sorghum MAPK genes

Gene ID	Gene name	5' UTR	3' UTR	Subcellular location
**Sobic.001G014200**	SbMAPK1	480	404	cyto: 10, chlo: 1, nucl: 1, plas: 1, cysk: 1
**Sobic.001G315700**	SbMAPK2	208	1068	chlo: 6, nucl: 5, cyto: 1, plas: 1, cysk: 1
**Sobic.001G410100**	SbMAPK3	278	283	cyto: 7, cysk: 4, nucl: 3
**Sobic.003G229400**	SbMAPK4	478	953	cyto: 8, nucl: 3, pero: 2, plas: 1
**Sobic.003G236000**	SbMAPK5	908	327	cyto: 8, nucl: 2, chlo: 1, mito: 1, pero: 1, cysk: 1
**Sobic.003G248100**	SbMAPK6	91	599	cyto: 8, chlo: 2, mito: 2, nucl: 1, cysk: 1
**Sobic.004G032800**	SbMAPK7	3616	308	nucl: 6, chlo: 3, cyto: 3, pero: 1, golg: 1
**Sobic.004G040200**	SbMAPK8	825	333	cyto: 8, nucl: 2, cysk: 2, mito: 1, golg: 1
**Sobic.004G270200**	SbMAPK9	162	484	nucl: 12, chlo: 1, cyto: 1
**Sobic.005G106600**	SbMAPK10	1304	402	cyto: 6, chlo: 3, nucl: 3, cysk: 2
**Sobic.007G046100**	SbMAPK11	403	416	chlo: 8, nucl: 1, cyto: 1, vacu: 1, E.R.: 1, pero: 1, cysk: 1
**Sobic.009G038900**	SbMAPK12	525	901	cyto: 7, nucl: 5, plas: 1, pero: 1
**Sobic.009G234300**	SbMAPK13	176	520	cyto: 6, cysk: 5, nucl: 3
**Sobic.009G242200**	SbMAPK14	191	381	chlo: 7, mito: 4.5, cyto_mito: 3, nucl: 2
**Sobic.009G246300**	SbMAPK15	842	203	cyto: 7, nucl: 5, vacu: 1, pero: 1
**Sobic.010G013200**	SbMAPK16	931	376	nucl: 9, chlo: 2, cyto: 2, cysk: 1
**Sobic.010G043200**	SbMAPK17	119	1001	nucl: 5, cyto: 4, cysk: 4, extr: 1
**Sobic.010G249500**	SbMAPK18	309	689	cyto: 8, nucl: 3, cysk: 2, golg: 1
**Sobic.010G257100**	SbMAPK19	349	540	cyto: 6, nucl: 3, chlo: 2, cysk: 2, mito: 1

Table S2. Distribution of the cis elements in the promoter regions (2000bp upstream) of the sorghum MAPK genes

**Motif name**	**Sorghum MAPK (SbMAPK)**																			**Functions**
	1	2	3	4	5	6	7	8	9	10	11	12	13	14	15	16	17	18	19	
ABRE	6	15	7	6	5	5	10	5	4	6	7	7	6	8	3	2	6	9	11	Involved in the abscisic acid responsiveness
GARE-motif	1	1	1	0	0	1	1	2	4	0	0	2	2	1	4	0	1	4	1	Gibberellin- responsive element
P-box	1	0	0	0	1	1	4	1	3	0	0	3	1	0	0	1	0	0	0	Gibberellin- responsive element
TCA-element	4	2	2	3	1	0	2	0	1	2	0	3	2	1	3	1	2	1	0	Salicylic acid responsiveness
ERE	1	0	0	0	2	2	1	0	0	1	0	2	1	0	0	2	3	0	0	ethylene rich motif
TGACG-motif	10	10	5	6	4	7	4	5	5	7	4	3	11	4	5	3	11	6	7	involved in the MeJA-responsiveness
CGTCA-motif	10	10	5	6	4	7	4	5	5	7	4	3	11	4	5	3	11	6	7	involved in the MeJA-responsiveness
AuxRR-core	1	0	0	0	2	0	0	1	0	0	2	0	0	0	0	0	2	0	0	auxin-responsive element
TGA-element	0	2	2	2	1	0	0	0	0	0	3	1	1	3	1	0	0	1	1	auxin-responsive element
W box	1	1	3	1	2	2	3	0	3	0	0	2	1	0	3	2	1	0	1	-
WRE3	5	6	1	2	1	6	3	0	1	6	3	4	5	1	3	2	6	0	4	-
WUN-motif	1	1	0	1	0	0	1	0	0	0	0	3	0	0	0	3	0	0	3	Wound related motif
TC-rich repeats	1	0	0	2	1	0	1	0	0	0	1	1	0	0	0	2	1	0	3	involved in defense and stress
CCAAT-box	3	4	1	2	1	1	1	1	2	0	1	1	2	0	2	1	0	3	1	Stress and defense inducible element
ARE	4	3	1	1	2	3	6	2	5	6	3	10	3	4	5	5	1	4	0	-
AAGAA-motif	0	1	1	6	1	2	5	0	1	1	1	2	3	4	7	1	3	0	3	-
as-1	10	10	5	6	4	7	4	5	5	7	4	3	11	4	5	1	11	6	7	-
DRE1	1	0	0	1	0	0	1	1	1	1	0	0	0	1	0	0	1	0	0	-
LTR	4	0	3	1	1	5	2	3	1	1	4	0	0	0	1	3	1	1	2	Involved in low temperature responsiveness
STRE	10	12	9	11	5	6	9	15	7	9	5	11	11	5	6	5	4	11	14	-
MBS	4	6	3	3	4	5	4	1	4	3	3	5	2	6	0	5	1	1	3	Drought inducible
MYB	10	8	9	11	4	10	9	8	10	11	7	9	8	15	11	7	3	8	6	-
MYC	12	3	6	8	7	10	21	6	7	11	9	11	10	7	8	6	13	9	3	-
Myb	4	8	4	6	11	8	5	1	4	5	5	7	4	9	4	6	2	2	5	Drought inducible
AAGAA-motif	0	1	1	6	1	2	5	0	1	1	1	2	3	4	7	1	3	0	3	-
Box 4	3	1	1	0	3	1	2	0	2	4	0	2	0	1	2	3	2	0	1	involved in light responsiveness

CCGTCC motif	4	2	6	3	2	3	0	3	2	3	3	4	2	2	6	0	2	7	2	-
CCGTCC-box	4	2	6	0	2	3	0	3	2	3	3	4	2	2	6	0	2	7	2	-
DRE core	1	7	6	0	2	1	1	1	1	2	3	2	1	1	3	3	3	3	5	-
CCAAT-box	3	4	1	2	1	1	1	1	2	0	1	1	2	0	2	1	0	3	1	MYBHv1 binding site
G-box	4	17	9	4	5	4	5	1	1	4	3	8	5	4	3		4	6	8	involved in light responsiveness

Table S3: the sorghum MAPK genes protein structure summary

Groups	Gene name	Template	Similarity	GMQE	QMEANDisCo Global	Description
Group A	SbMAPK3	6dtl.1.A	74.17%	0.82	0.82 ± 0.05	Mitogen-activated protein kinase 6
	SbMAPK17	6dtl.1.A	89.47%	0.82	0.82 ± 0.05	Mitogen-activated protein kinase 6
Group B	SbMPK2	7w5c.1.A	83.10%	0.84	0.84 ± 0.05	Mitogen-activated protein kinase 4
	SbMPK11	7w5c.1.A	81.97%	0.84	0.84 ± 0.05	Mitogen-activated protein kinase 4
Group C	SbMPK8	6dtl.1.A	59.36%	0.78	0.78 ± 0.05	Mitogen-activated protein kinase 6
	SbMPK18	6dtl.1.A	58.60%	0.78	0.78 ± 0.05	Mitogen-activated protein kinase 6
Group D	SbMPK10	6dtl.1.A	47.95%	0.51	0.72 ± 0.05	Mitogen-activated protein kinase 1
	SbMPK12	5o7i.1.A	50.44%	0.45	0.73 ± 0.05	Mitogen-activated protein kinase 7
	SbMPK7	5o7i.1.A	47.67%	0.57	0.73 ± 0.05	Mitogen-activated protein kinase 7
	SbMPK19	5o7i.1.A	47.65%	0.46	0.72 ± 0.05	Mitogen-activated protein kinase 7
	SbMPK5	5o7i.1.A	47.38%	0.56	0.73 ± 0.05	Mitogen-activated protein kinase 7
	SbMPK14	4ic7.1.A	46.32%	0.44	0.65 ± 0.05	Mitogen-activated protein kinase 7
	SbMPK4	5o7i.1.A	46.32%	0.42	0.73 ± 0.05	Mitogen-activated protein kinase 7
	SbMPK15	6dtl.1.A	47.08%	0.42	0.72 ± 0.05	Mitogen-activated protein kinase 1
	SbMPK6	4qtb.1.A	45.30%	0.44	0.72 ± 0.05	Mitogen-activated protein kinase 3
	SbMPK13	8rlx.1.A	45.38%	0.43	0.72 ± 0.05	Mitogen-activated protein kinase 1
Group E	SbMPK1	5o7i.1.A	47.66%	0.46	0.73 ± 0.05	Mitogen-activated protein kinase 7
	SbMPK9	2iw8.1.A	39.72%	0.47	0.70 ± 0.05	Cell division protein kinase 2
	SbMPK16	9gla.1.A	41.72%	0.45	0.70 ± 0.05	Cyclin-dependent kinase 2

Table S4: Nomenclature of MAPKs in Sorghum and comparison with those in Brachypodium and Arabidopsis.

Sorghum gene name	Sorghum gene ID	Brachypodium gene name	Brachypodium gene ID	Arabidopsis gene name	Arabidopsis gene ID
SbMAPK1	Sobic.001G014200	BdMAPK3	Bradi1g65810	AtMAPK1	At1g10210
SbMAPK2	Sobic.001G315700	BdMAPK4	Bradi3g32000	AtMAPK2	At1g59580
SbMAPK3	Sobic.001G410100	BdMAPK6	Bradi1g49100	AtMAPK3	At3g45640
SbMAPK4	Sobic.003G229400	BdMAPK7-1	Bradi1g34030	AtMAPK4	At4g01370
SbMAPK5	Sobic.003G236000	BdMAPK7-2	Bradi4g24912	AtMAPK5	At4g11330
SbMAPK6	Sobic.003G248100	BdMAPK11	Bradi3g16560	AtMAPK6	At2g43790
SbMAPK7	Sobic.004G032800	BdMAPK14	Bradi3g03780	AtMAPK7	At2g18170
SbMAPK8	Sobic.004G040200	BdMAPK16	Bradi2g36470	AtMAPK8	At1g18150
SbMAPK9	Sobic.004G270200	BdMAPK17	Bradi1g34700	AtMAPK9	At3g18040
SbMAPK10	Sobic.005G106600	BdMAPK20-1	Bradi2g44350	AtMAPK10	At3g59790
SbMAPK11	Sobic.007G046100	BdMAPK20-2	Bradi2g15317	AtMAPK11	At1g01560
SbMAPK12	Sobic.009G038900	BdMAPK20-3	Bradi1g41780	AtMAPK12	At2g46070
SbMAPK13	Sobic.009G234300	BdMAPK20-4	Bradi2g45870	AtMAPK13	At1g07880
SbMAPK14	Sobic.009G242200	BdMAPK20-5	Bradi2g16337	AtMAPK14	At4g36450
SbMAPK15	Sobic.009G246300	BdMAPK21-1	Bradi2g15620	AtMAPK15	At1g73670
SbMAPK16	Sobic.010G013200	BdMAPK21-2	Bradi2g45010	AtMAPK16	At5g19010
SbMAPK17	Sobic.010G043200			AtMAPK17	At2g01450
SbMAPK18	Sobic.010G249500			AtMAPK18	At1g53510
SbMAPK19	Sobic.010G257100			AtMAPK19	At3g14720
				AtMAPK20	At2g42880

Table S5: Nomenclature of MAPKs in Maize, Poplar, and Foxtail millet.

Maize gene name	Maize gene ID	Poplar gene name	Poplar gene ID	Foxtail millet gene name	Foxtail millet gene ID
ZmMAPK1	Zm00001d014658	PtMAPK1	Potri.002G032100	SiMPK1	Seita.9G444100
ZmMAPK2	Zm00001d015269	PtMAPK2	Potri.005G231100	SiMPK2	Seita.9G344000
ZmMAPK3-1	GRMZM2G053987	PtMAPK3-1	Potri.009G066100	SiMPK3	Seita.4G069900
ZmMAPK3-1	Zm00001d047758	PtMAPK3-2	Potri.001G271700	SiMPK4	Seita.4G243400
ZmMAPK3-2	Zm00001d028711	PtMAPK4	Potri.014G088500	SiMPK5	Seita.6G056700
ZmMAPK4	Zm00001d047349	PtMAPK5-1	Potri.001G099900	SiMPK6	Seita.1G089400
ZmMAPK6-1	Zm00001d036215	PtMAPK5-2	Potri.003G131800	SiMPK7	Seita.8G104500
ZmMAPK6-2	Zm00001d045310	PtMAPK6-1	Potri.007G139800	SiMPK8	Seita.4G273900
ZmMAPK8	Zm00001d011465	PtMAPK6-2	Potri.017G010200	SiMPK9	Seita.1G095300
ZmMAPK12	Zm00001d024568	PtMAPK7	Potri.005G119500	SiMPK10	Seita.5G235700
ZmMAPK15	Zm00001d039105	PtMAPK9-1	Potri.012G048600	SiMPK11	Seita.3G058000
ZmMAPK16	Zm00001d024807	PtMAPK9-2	Potri.015G040300	SiMPK12	Seita.J012000
ZmMAPK17-1	Zm00001d053997	PtMAPK11	Potri.002G162500	SiMPK13	Seita.5G261300
ZmMAPK17-2	Zm00001d036448	PtMAPK14	Potri.007G020100	SiMPK14	Seita.3G145200
ZmMAPK17-3	Zm00001d014726	PtMAPK16-1	Potri.008G200800	SiMPK15	Seita.3G136100
ZmMAPK18-1	Zm00001d043954	PtMAPK16-2	Potri.010G029700	SiMPK16	Seita.5G241700
ZmMAPK18-2	Zm00001d011525	PtMAPK17	Potri.010G112200		
ZmMAPK19	Zm00001d009587	PtMAPK18	Potri.011G102500		
ZmMAPK20-1	Zm00001d039141	PtMAPK19	Potri.001G381300		
ZmMAPK20-2	Zm00001d011426	PtMAPK20-1	Potri.005G201800		
		PtMAPK20-2	Potri.002G059900		

Table S6: Nomenclature of MAPK outgroup.

Outgroup gene names	Outgroup gene IDs
HsCDK1 (*Homo sapiens* cyclin-dependent kinase 1)	Ensembl ID: ENSP00000378699
HsCDK17 (*Homo sapiens* cyclin-dependent kinase 17)	Ensembl ID: ENSG00000059758 ENSP00000261211
Saccharomyces cerevisiae mitogen-activated serine/threonine-protein kinase FUS3 (FUS3)	NP_009537.1
Chlamydomonas reinhardtii cyclin-dependent kinases (CrCDK)	XP_001690852

Table S7: Nine designed primers.

Gene ID	Gene name	Primers
Sobic.001G014200	SbMAPK1-F	AGGCAAGTTCCTTTCTCTGAAG
	SbMAPK1-R	CGCGGTGGAAGAATCCATTA
Sobic.001G410100	SbMAPK3-F	CAGAGGATCACAGTTGAAGAGG
	SbMAPK3-R	GCTCGAAGTCGAACGAGAAT
Sobic.003G229400	SbMAPK4-F	GTCCACACTGGAGAGAAAGTAG
	SbMAPK4-R	GGAGTCTCAGAAGCTTGATCTC
Sobic.003G236000	SbMAPK5-F	TCGAAGAAAGTGCTGCTAAGG
	SbMAPK5-R	GTCGTCGCTGCTGTCATTTA
Sobic.004G032800	SbMAPK7-F	TTCCTGGGAGGAATGTTGTG
	SbMAPK7-R	GGCCTTATCACTGCGAATCT
Sobic.004G270200	SbMAPK9-F	TTGACACTCCATCCTCTCTTTC
	SbMAPK9-R	CTTGAGGCCAAGATTGTTCATC
Sobic.009G234300	SbMAPK13-F	CGGACTATGTTGCTACCAGATG
	SbMAPK13-R	CAACCGATGCTCCACATATCA
Sobic.009G242200	SbMAPK14-F	CTCCTTCGAGGAGGGAATTTAG
	SbMAPK14-R	GTGATGCTCTGC TGTGAGAT
Sobic.009G246300	SbMAPK15-F	TGACTTGACCAAGGAGCATTAC
	SbMAPK15-R	GCTTCAGGTCACGGTGATAAA
